# Identification of an optimal threshold to define oliguria in critically ill patients: an observational study

**DOI:** 10.1186/s13054-023-04505-7

**Published:** 2023-05-30

**Authors:** Nathan Axel Bianchi, Marco Altarelli, Céline Monard, Tatiana Kelevina, Aziz Chaouch, Antoine Guillaume Schneider

**Affiliations:** 1 Adult Intensive Care Unit, Centre Hospitalier Universitaire Vaudois (CHUV), 46, Avenue du Bugnon, 1011 Lausanne, Switzerland; 2 Faculty of Biology and Medicine, University of Lausanne, Lausanne, Switzerland; 3 Department of Epidemiology and Health Systems, Center for Primary Care and Public Health (Unisanté), University of Lausanne, Lausanne, Switzerland

**Keywords:** Acute kidney injury, Urinary output, Oliguria, Thresholds, ICU mortality, Critical illness

## Abstract

**Background:**

The relevance of current consensus threshold to define oliguria has been challenged by small observational studies. We aimed to determine the optimal threshold to define oliguria in critically-ill patients.

**Methods:**

Cohort study including adult patients admitted within a multi-disciplinary intensive care unit between January 1st 2010 and June 15th 2020. Patients on chronic dialysis or who declined consent were excluded. We extracted hourly urinary output (UO) measurements along with patient’s characteristics from electronic medical records and 90-day mortality from the Swiss national death registry. We randomly split our data into a training (80%) and a validation (20%) set. In the training set, we developed multivariable models to assess the relationship between 90-day mortality and the minimum average UO calculated over time windows of 3, 6, 12 and 24 h. Optimal thresholds were determined by visually identifying cut-off values for the minimum average UO below which predicted mortality increased substantially. We tested models’ discrimination and calibration on the entire validation set as well as on a subset of patients with oliguria according to proposed thresholds.

**Results:**

Among the 15,500 patients included in this analysis (training set: 12,440, validation set: 3110), 73.0% (95% CI [72.3–73.8]) presented an episode of oliguria as defined by consensus criteria (UO < 0.5 ml/kg/h for 6 h). Our models had excellent (AUC > 85% for all time windows) discrimination and calibration. The relationship between minimum average UO and predicted 90-day mortality was nonlinear with an inflexion point at 0.2 ml/kg/h for 3 and 6 h windows and 0.3 ml/kg/h for 12 and 24 h windows. Considering a threshold of < 0.2 ml/kg/h over 6 h, the proportion of patients with an episode of oliguria decreased substantially to 24.7% (95% CI [24.0–25.4]). Contrary to consensus definition, this threshold identified a population with a higher predicted 90-day mortality.

**Conclusions:**

The widely used cut-off for oliguria of 0.5 ml/kg/h over 6 h may be too conservative. A cut-off of 0.2 ml/kg/h over 3 or 6 h is supported by the data and should be considered in further definitions of oliguria.

**Supplementary Information:**

The online version contains supplementary material available at 10.1186/s13054-023-04505-7.

## Background

Urinary output (UO) is the most obvious and accessible window to renal function in the intensive care unit (ICU). It is a major component of acute kidney injury (AKI) diagnosis and staging [1]. Low UO (oliguria) is associated with 90-day mortality irrespective of changes in serum creatinine (sCr) [2–4]. However, the interpretation of oliguria remains a daily challenge for clinicians throughout the world who struggle to differentiate a physiological adaptation to stress from kidney damage. According to consensus definition, a urinary output of less than 0.5 ml/kg/h for a minimal duration of 6 h defines AKI[1]. This threshold is based on old physiological data demonstrating that urine was maximally concentrated when its volume output fell below 0.35 ml/min (21 ml/h) [5].

The clinical relevance of this definition, however, has recently been challenged [6–9]. In a series of > 3500 patients, intra-operative episodes of oliguria defined by a UO between 0.3 and 0.5 ml/kg/h were not associated with post-operative AKI but only those defined by a UO < 0.3 ml/kg/h [10]. In 239 critically ill patients, oliguria, defined by a cut-off of 0.5 ml/kg/h for > 4 h, had a very low positive predictive value (11%) for AKI (defined as an increased serum creatinine) [11]. In 725 critically ill patients, a threshold of 0.3 ml/kg/h for 6 h outperformed the current definition to predict mortality or the need for dialysis [6]. Those findings were confirmed in a larger cohort study conducted in Finland, where, compared to standard thresholds, stricter (< 0.3 ml/kg/h for 6 h or < 0.1 ml/kg/h for 3 h) cut-offs increased the predictive values of oliguria for 90-day mortality [12].

Hence, the current definition of oliguria appears too conservative. However, the best cut-off to define oliguria remains to be determined. We aimed to define the best time weighted intensity cut-offs for UO to predict 90-day mortality in critically ill patients. Accordingly, we designed a retrospective observational study to assess the association between the minimum average UO and 90-day mortality, to identify potential cut-offs in this relationship and to assess their clinical relevance.

## Methods

### Design, setting, participants, data sources and statistics

For this observational study, we have used a high-resolution cohort described in details elsewhere [2]. Briefly, we collected data from all adult (> 18 years old) patients admitted to our tertiary ICU between January 1st 2010 and June 15th 2020. We excluded patients with documented or expressed wishes of non-participation to clinical research, those with end-stage renal disease (ESRD), whose stay lasted for less than 6 h or with missing outcome or AKI defining data. For each patient, we considered only the first eligible ICU admission. Data were extracted from electronic medical records [Metavision®(IMD Soft, Tel Aviv, Israel) and Soarian® (Cerner, North Kansas City, USA)]. In particular, we collected baseline characteristics, comorbidities, pre-admission weight, illness severity scores and hourly UO measurements. The primary outcome was 90-day mortality. This outcome was assessed by cross-referencing our dataset with the Swiss national death registry.

Continuous data are reported as mean (standard deviation, SD) or median (interquartile range, IQR) according to underlying data distribution. Categorical variables are expressed as number (percentage). All statistical analyses and modeling were carried out in R version 4.1.2 [13]. The level of statistical significance was set at 5%.

#### Average standardized urinary output calculation

We standardized hourly UO (ml/h) (${v}_{is}$) by pre-admission body weight (${w}_{i}$) when available (missing values were imputed using multiple imputations see below). We then used a sliding window to calculate the average standardized urinary output (${\overline{v}}_{it}$) of a patient *i* over $d$ hours preceding time* t*, such that

$${\overline{v}}_{it}\left(d\right)=\frac{1}{d}{\sum }_{s=t-d+1}^{t}\frac{{v}_{is}}{{w}_{i}}$$

For convenience, we restricted the analysis to sliding windows of width $d=\left\{3,6, 12,24\right\}$ hours. Note that ${\overline{v}}_{it}\left(d\right)$ cannot be calculated when $t<d$. We then calculated the minimum value among all moving averages of width *d* that can be computed over the whole ICU stay of each patient, that is

$${u}_{i}\left(d\right)=\underset{t\ge d}{\mathrm{min}}\left({\overline{v}}_{it}\left(d\right)\right)$$

Hence, ${u}_{i}\left(d\right)$ corresponds to the minimal average UO that patient *i* experienced over a period of *d* hours during his ICU stay.

Of note, we considered pre-admission body weight when available.

#### Modeling 90-day mortality

Logistic regression was used to predict 90-day mortality as a function of the minimum average urine output of patients, separately for medical, scheduled surgical and unscheduled surgical admissions. Within each admission type, control variables included patient’s age at ICU admission, SAPS II score (corrected to not account for daily UO) and Charlson comorbidities index. All predictors were continuous and flexibly modeled using penalized thin plate regression splines within the framework of generalized additive models [14, 15]. Alternative candidate models also included smooth terms for the interaction between minimal average UO and other continuous predictors. A model which did not include minimum average UO as predictor (“base model”) was also fitted for comparative purposes. The model featuring the highest overall calibration performance (i.e., lowest mean squared error of prediction) across all time windows was selected using tenfold cross-validation. For each value of *d*, predicted 90-day mortality was plotted as a function of the minimal average UO for fixed covariate patterns (e.g., corresponding to median values of control variables). This allowed visual identification of suitable oliguria thresholds, i.e., UO thresholds below which mortality increases substantially, and compare these with thresholds used in current practice.

Data were randomly split into a training (80%) and a validation set (20%). Training data were used to develop and fit prognostic models (including selection of the best model using tenfold cross-validation). Validation data were exclusively used to evaluate the final models’ discrimination and calibration properties. Discrimination was assessed using the area under the receiver operator characteristic curve (AU-ROC). Calibration was assessed using Hosmer–Lemeshow test [16] and calibration belt [17]. Discrimination and calibration performances were assessed on all validation data as well as on subsets of patients whose minimum average urine output fell below proposed thresholds for oliguria.

Finally, we performed sensitivity analyses on the training set, to assess the influence of body weight, gender, illness severity and time periods on our results.

#### Handling missing covariate data

Missing covariate values were imputed using multiple imputations [18] with a total of 50 complete datasets being reconstructed. Multiple imputations were carried out separately within training and validation sets. Additional variables such as gender, body weight, baseline creatinine, need for noradrenaline were used in the imputation process. Note that when body weight ${w}_{i}$ was missing, imputed weights were used to calculate imputed values for ${\overline{v}}_{it}\left(d\right)$ and thus ${u}_{i}\left(d\right)$ as well. Rubin rules [18] were used to pool predicted mortalities, AU-ROC estimates and/or calibration results obtained within each complete dataset.

Urinary output is collected manually on an hourly basis by ICU nurses. The management of missing UO values is described in details in the Additional file 1.

### Ethics

This study was approved by the Ethics Committee Vaud (CER-VD 2017-00008, Lausanne, Switzerland). In accordance with the Swiss Federal Act on Research involving Human Beings (article 34) [19], retrospective utilization of non-genetic health-related personal data was permitted, provided that the patient (or its legal representative) had not expressed wishes of non-participating to clinical research. This study followed “The Strengthening the Reporting of Observational Studies in Epidemiology” (STROBE) guidelines for reporting observational studies.

## Results

### Population

Among the 18,314 patients admitted to our ICU during the study period. 2584 were excluded: 1116 declined consent for data utilization for research, 84 were under 18 years old, 747 had an ICU stay of less than 6 h, 217 had ESRD, 45 had no administrative data available, 305 had incomplete UO or sCr data and 70 had unknown 90-day mortality status. Therefore, our main analysis included 15,550 patients (Table 1). Patients were predominantly male (10,283, 66.1%) with a median (IQR) age of 65 (53.0–75.0) years, a median body weight of 75.0 (65.0–87.0) kg, a median Charlson score of 4 (2.0–6.0) and a median corrected SAPS II score of 37 (28–48). Among those, 73.0% (95% CI [72.3–73.8]) presented a minimal average UO below 0.5 ml/kg/h for 6 h and therefore fulfilled criteria for oliguria according to consensus definition. Clinical outcomes and missing values are reported in the Additional file 1 (Additional file 1: Tables S1 and S2). Pre-admission body weight was not available and had to be imputed in 3188 (20.5%) patients (Cf. multiple imputations above).

**Table 1:** Patients and ICU stay characteristics

	All patients (*n* = 15,550)	Training set (*n* = 12,440)	Validation set (*n* = 3110)	*P* value
*Demographics*				
Male, *n* (%)	10,283 (66.1)	8221 (66.1)	2062 (66.3)	0.84
Female, *n* (%)				
Age at ICU admission, median (IQR), years	65.0 (53.0, 75.0)	65.0 (53.0, 75.0)	65.0 (53.0, 75.0)	0.50
Pre-admission body weight, median (IQR), kg	75.0 (65.0, 87.0)	75.0 (65.0, 87.0)	75.0 (65.0, 87.0)	0.37
Retrieved from medical records	12,362 (79.5)	9897 (79.6)	2465 (79.3)	0.84
Imputed (multiple imputations)	3188 (20.5)	2543 (20.4)	645 (20.7)	
Baseline creatinine, median (IQR), μmol/L	69.0 (54.0, 90.0)	69.0 (53.0, 90.0)	70.0 (54.0, 91.0)	0.07
*Comorbidities*				
Charlson score, median (IQR)	4.0 (2.0, 6.0)	4.0 (2.0, 6.0)	4.0 (2.0, 6.0)	0.19
Chronic kidney disease, *n* (%)	1610 (10.4)	1255 (10.1)	355 (11.5)	0.03
Hypertension, *n* (%)	7286 (47.0)	5804 (46.8)	1482 (47.8)	0.33
Diabetes, *n* (%)	2926 (18.9)	2307 (18.6)	619 (20.0)	0.09
Heart failure, *n* (%)	3454 (22.3)	2747 (22.2)	707 (22.8)	0.45
Chronic obstructive pulmonary disease, *n* (%)	1806 (11.7)	1455 (11.7)	351 (11.3)	0.54
Myocardial infarction, *n* (%)	5430 (35.0)	4357 (35.1)	1073 (34.6)	0.60
Chronic liver disease, *n* (%)	1188 (7.7)	957 (7.7)	231 (7.5)	0.65
Cancer, *n* (%)	2900 (18.7)	2312 (18.6)	588 (19.0)	0.70
*ICU admission characteristics*				
Type of admission				0.63
Medical	6534 (42.8)	5248 (43.0)	1286 (42.1)	
Surgical scheduled	4374 (28.7)	3487 (28.6)	887 (29.0)	
Surgical unscheduled	4345 (28.5)	3461 (28.4)	884 (28.9)	
Main ICU diagnosis				0.08
Cardiovascular surgery	3505 (23.1)	2812 (23.1)	693 (22.7)	
Other heart disease	1963 (12.9)	1602 (13.2)	361 (11.8)	
Septic shock	1762 (11.6)	1387 (11.4)	375 (12.3)	
Neurologic	1614 (10.6)	1293 (10.6)	321 (10.5)	
Trauma	1236 (8.1)	958 (7.9)	278 (9.1)	
Acute respiratory insufficiency	1313 (8.6)	1061 (8.7)	252 (8.3)	
Cardiopulmonary arrest	897 (5.9)	731 (6.0)	166 (5.4)	
Other	2908 (19.1)	2306 (19.0)	602 (19.8)	
sCr at ICU admission, median (IQR), μmol/L	83.0 (65.0, 112.0)	83.0 (65.0, 112.0)	83.0 (65.0, 114.0)	0.32
Mechanical ventilation within 24 h of ICU admission	9152 (58.9)	7346 (59.1)	1806 (58.1)	0.33
Noradrenaline within 24 h of ICU admission	10,702 (68.8)	8547 (68.7)	2155 (69.3)	0.54
Modified SAPS II score^a^, median (IQR)	37.0 (28.0, 48.0)	37.0 (28.0, 48.0)	37.0 (28.0, 48.0)	0.97

Presented percentage exclude missing values. *P* value provided is for comparison between patients in the training set and the validation set. Number of missing values are reported in Additional file 1

ICU, intensive care unit; sCr, serum creatinine; SAPS, simplified acute physiology score

^a^Modified SAPS II does not include points (0 to + 11) for urine output

### Relationship between UO and 90-day mortality

The training and validation sets included data from 12,440 and 3110 patients, respectively (Table 1).

**Fig. 1:**
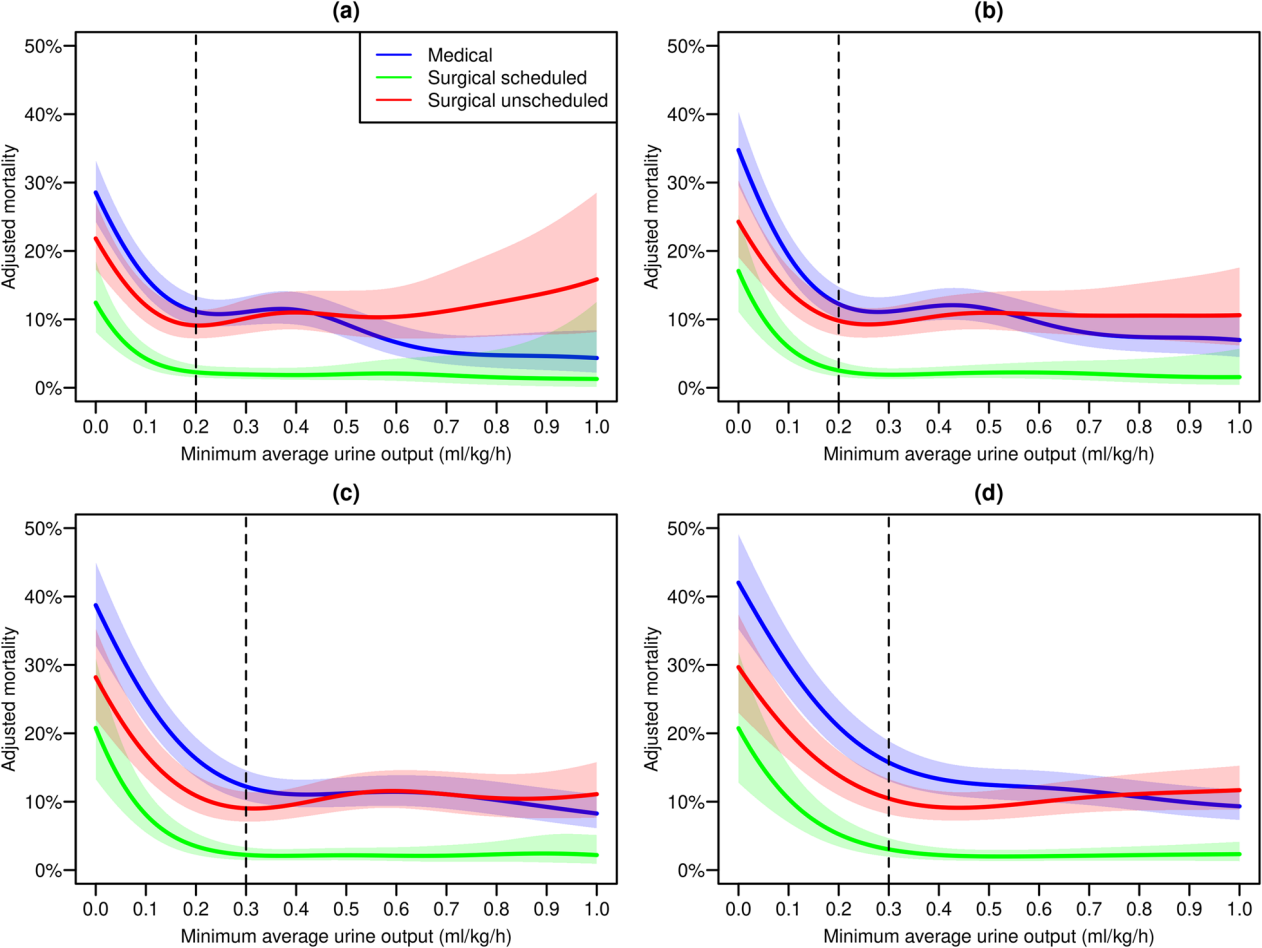
Adjusted* 90-day mortality as a function of the minimum average urine output for time windows of 3 h (**a**), 6 h (**b**), 12 h (**c**) and 24 h (**d**). Data is stratified by type of admission (medical and scheduled/unscheduled surgical admissions). Colored areas refer to 95% confidence intervals around the regression lines. Vertical dashed lines refer to thresholds below which the adjusted mortality increases substantially. *Predictions are carried out for a fictive patient with continuous predictors fixed at their median value (i.e., 65 years old at ICU admission, corrected SAPS II score of 37 and Charlson index of 4)

Figure 1 illustrates the dependence between the minimum average UO (computed over time windows of 3, 6, 12 or 24 h) and 90-day predicted mortality in the different categories of admissions. Predicted mortality is estimated for median values of age, corrected SAPS II score and Charlson index. This figure suggests that adjusted 90-day mortality increases substantially in all categories of admission when the minimum average urine output is below 0.2 ml/kg/h over 3 or 6 h, or below 0.3 ml/kg/h over 12 or 24 h. Unadjusted data are presented in Additional file 1: Figure S1.

Similar associations and thresholds between minimum average UO and mortality were observed when using the same model to predict 30-day mortality (Additional file 1: Figure S2). Similarly, in sensitivity analyses, extreme patients’ weights (< 43 kg or > 130 kg; Additional file 1: Figure S3), gender (Additional file 1: Figures S4 and S5), SAPS II scores (Additional file 1: Figures S6 and S7) and time periods (Additional file 1: Figures S8, S9 and S10) did not appear to influence these results.

### Proposal for a new definition of oliguria

**Fig. 2:**
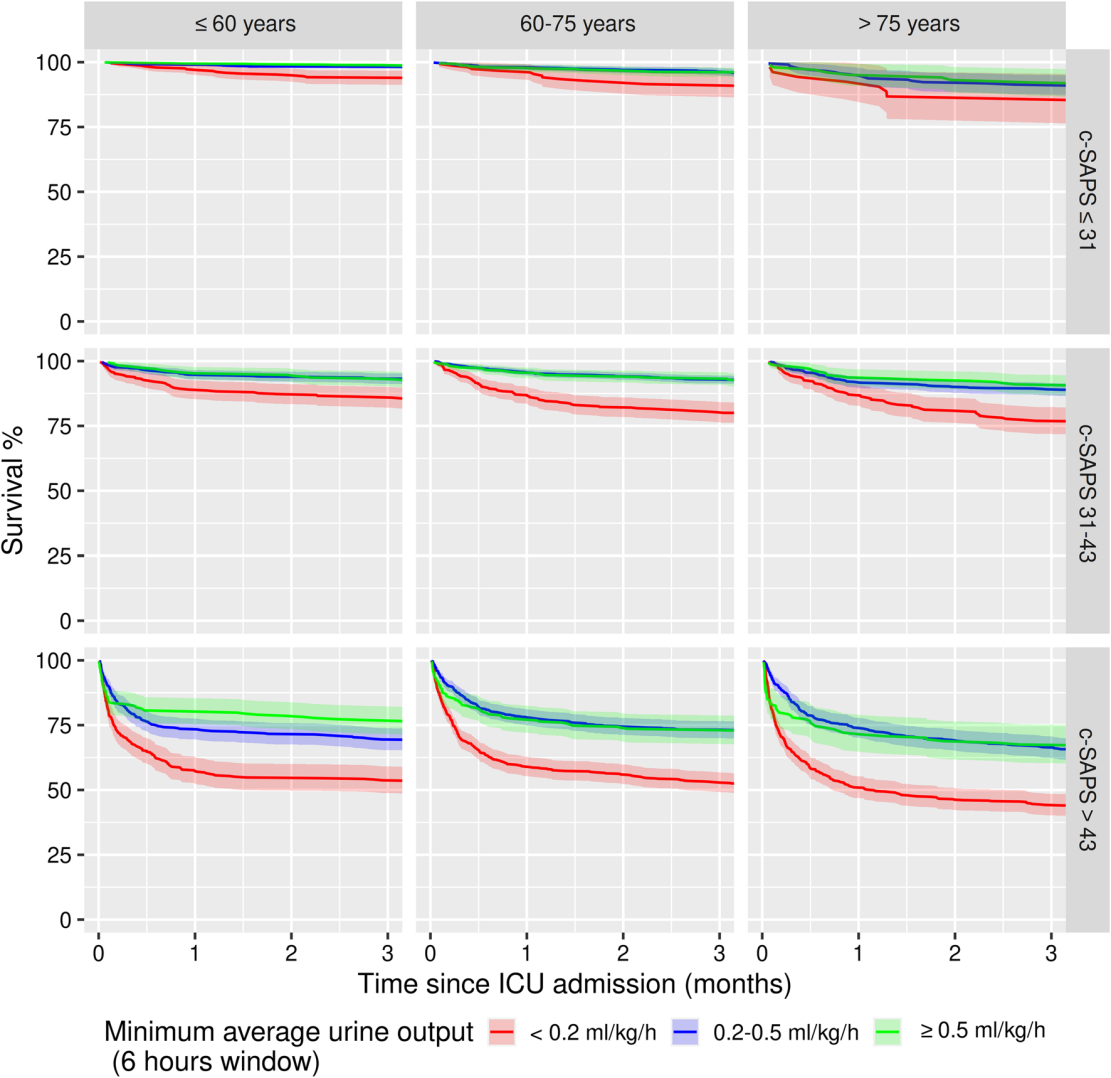
Kaplan–Meier 3-month survival curves according to minimum average urinary output (6 h windows). Data is stratified by tertiles of age and corrected SAPS score. Analyses are restricted to patients with available body weight (no imputation) *n* = 12,658. *Urine output corresponds to the minimum average urinary output over a period of 6 h

Based on these findings, we propose to consider a minimum average UO of < 0.2 ml/kg/h for 6 h as the new threshold to define oliguria. In our cohort, 24.7% [95% CI 24.0–25.4] of the patients fulfilled these revised criteria for oliguria (vs 73.0% [95% CI 72.3–73.8]) for consensus criteria). Unlike consensus criteria, this stricter definition is significantly associated with a higher 90-day adjusted mortality (OR 1.98 [95% CI 1.57–2.49] vs 1.27 [95% CI 0.95–1.70]). As shown in Fig. 2, patients with a minimum average UO of < 0.2 ml/kg/h for 6 h had a lower 3-month survival compared to other groups of patients throughout categories of illness severity and age, and this persisted at 12 months, (Additional file 1: Figure S11).

### Confirmatory analyses

Our prognostic models achieved good discrimination on validation data, with AU-ROC exceeding 85% for all time windows (Table 2). However, prior univariate analyses identified the corrected SAPS II score as the strongest predictor of 90-day mortality, with AU-ROC already reaching 82%. As a consequence, only minor improvements in discrimination were observed when accounting for the minimal average urine output in prognostic models over the base model (Table 2).

**Table 2:** Area under the receiver operating characteristic curve (AU-ROC) for all patients in the validation set as well as for validation patients complying with suggest oliguria thresholds

Time window	Suggested oliguria threshold (ml/kg/h)	All validation patients		Validation patients with oliguria	
		Base model	Full model	Base model	Full model
3 h	< 0.2	85.3	86.1	82.1	83.0
6 h	< 0.2	85.3	85.9	80.0	81.5
12 h	< 0.3	85.3	86.1	82.5	83.7
24 h	< 0.3	85.3	86.1	79.8	82.0

Compared with base model that does not include minimum average urine output as predictor, the full model demonstrates only minimal increase in prediction as illustrated by minor improvement in AU-ROC across all time windows

**Fig. 3:**
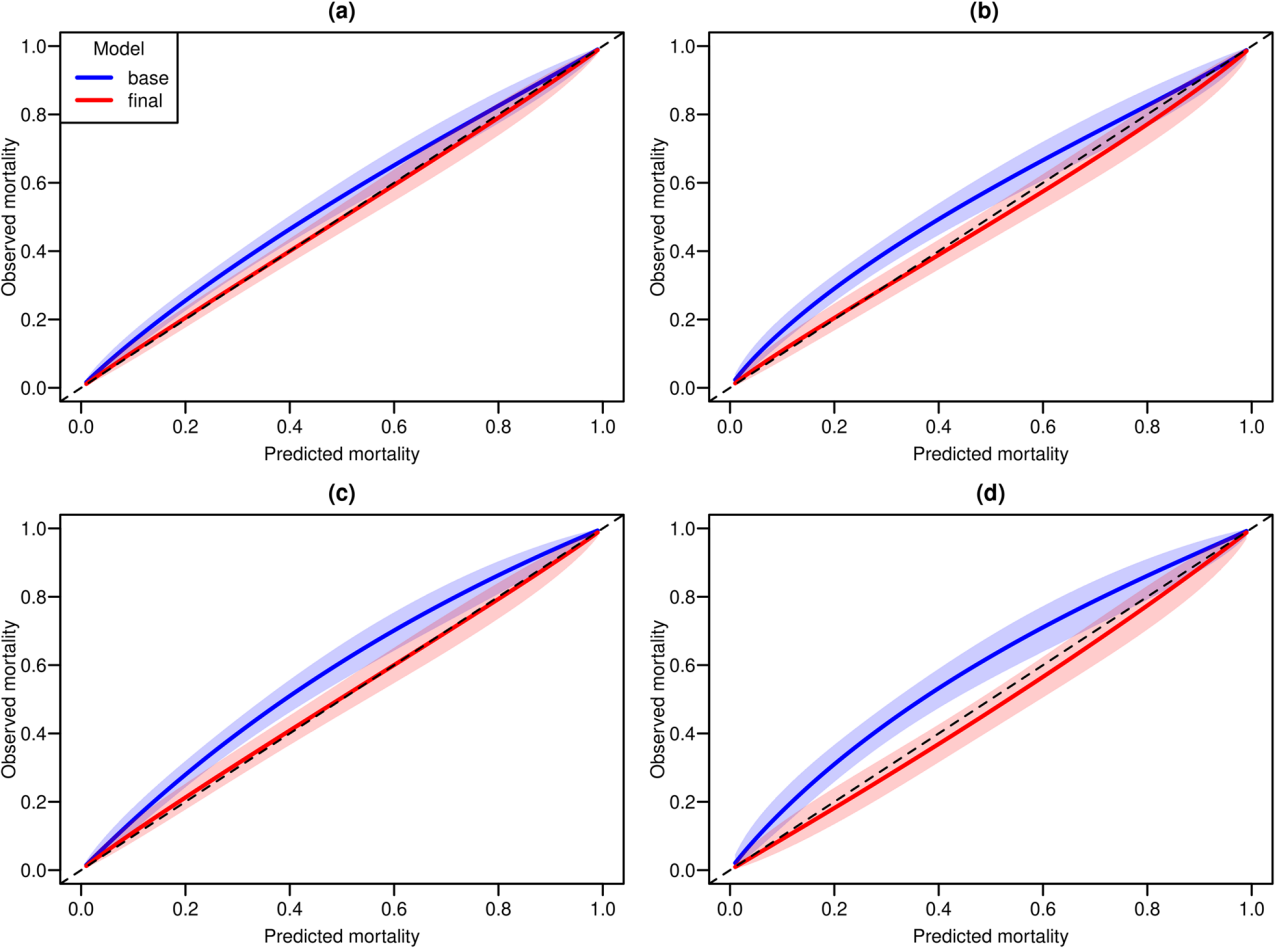
Calibration belts for patients in the validation set complying with the proposed oliguria definitions (UO < 0.2 ml/kg/h) for time windows of 3 h (**a**), 6 h (**b**), 12 h (**c**) and 24 h (**d**). Blue: base model (unadjusted for minimum average UO); red final model (adjusted for minimum average UO). Colored areas refer to 95% pointwise confidence intervals for observed mortality

When considering all validation data, calibration performance was very good (Additional file 1: Figure S12) both for the base model and the model including minimal average UO as a predictor of mortality. On the other hand, calibration belts presented in Fig. 3 suggest that, when restricting the attention to patients complying with the proposed oliguria definitions, the calibration of predicted mortalities improved substantially when accounting for the minimal average urine output. This was further supported by statistically significant (*p* < 0.001) results returned by Hosmer–Lemeshow tests on validation data for all models that omitted urine output from the list of predictors of mortality, suggesting that predicted probabilities of death derived from such models were miscalibrated in patients with oliguria. On the other hand, models which included UO as a predictor of mortality showed no evidence of miscalibration in these patients, with *p* values > 0.05 for 3, 6, 12 and 24 h time windows.

## Discussion

### Summary of key findings

We conducted a large observational study including more than 15,000 patients admitted to a tertiary ICU during a 10-year period. We assessed the association between minimum average UO (calculated over time windows of 3, 6, 12 and 24 h) and 90-day mortality after controlling for age on admission, comorbidities and illness severity. When assessed on validation data, prognostic models including minimum average UO as a predictor of 90-day mortality resulted in good calibration and discrimination. We observed a nonlinear relationship between minimum average UO and 90-day mortality. Our results suggest that the widely used cut-off for oliguria of 0.5 ml/kg/h over 6 or 12 h may be too conservative. We found that values of 0.2 ml/kg/h (3 and 6 h time-windows) and 0.3 ml/kg/h (12 and 24 h time-windows) had higher prognostic implications. These values were similar for patients admitted for medical or surgical reasons.

### Comparisons with previous studies

Only a handful of studies have evaluated the relationship between oliguria and clinical outcomes. Two have considered the occurrence of AKI (defined as a sCr rise) as an outcome [10, 11]. In those studies, oliguria, defined as a UO < 0.5 ml/kg/h for 6 h, only had a weak association with AKI and very limited positive predictive value. Intrinsically, however, this design implies that only a rise in sCr could define *true* AKI while UO only represents either an associated finding or a biomarker. This is in contradiction with recent data which suggest that oliguria might be associated with mortality irrespective of sCr rise [2, 3]. Therefore, patients’ centered outcomes such as mortality or need for renal replacement therapy (RRT) appear more suitable to assess the relevance of oliguria.

Our data are in agreement with other smaller observational studies conducted in other health systems such as New Zealand or Finland [6, 12]. Ralib et al. have determined optimal UO thresholds for prediction of mortality or the need for RRT for time windows of 1 to 12 h. These cut-offs were derived from AUC-ROC curves at a univariate level. For the 3- 6- and 12-h time windows, identified cut-offs were, respectively, 0.2, 0.3 and 0.5 ml/kg/h. Our analyses, based on a much larger dataset and modeling UO as a continuous predictor with smooth functional forms while accounting for age, comorbidities and severity score, lead to slightly different values particularly at 12 h. However, we confirm the authors’ impression that current definition of oliguria is too liberal. In an analysis of the FINNAKI cohort, episodes of severe (< 0.1 ml/kg/h) oliguria lasting more than 3 consecutive hours were independently associated with the development of sCr-AKI or RRT. In this analysis, consistent with our data, oliguria defined as a UO between 0.3 and 0.5 ml/kg/h for 6 h, was not significantly associated with adjusted 90-day mortality (OR 1.65 [95% CI 1.0–2.72]). The shortest periods of consecutive oliguria associated with an increased risk for 90-day mortality on multivariate analyses were 6 h (for 0.1 to 0.3 ml/kg/h) and 3 h (for < 0.1 ml/kg/h).

In contrast, an analysis from the MIMIC-2 cohort suggested that mortality increased rapidly as UO decreased < 0.5 ml/kg/h [20]. These analyses used multivariate logistic regression analysis and contour plots. The difference in appreciation with our study is most likely explained by different modeling strategies for UO. Indeed, the authors have dichotomized UO whereas it was treated as a continuous predictor in the present study. Due to the nonlinear effect of UO on 90-day mortality (observed in our data), a dichotomous treatment of UO would likely lead to an overestimation of the optimal oliguria threshold. Indeed, a dichotomous threshold of 0.5 ml/kg/h to define oliguria would already lead to a difference in estimated mortality between oliguric and non-oliguric patients while this mortality signal is only driven by the fraction of patients with UO < 0.2 ml/kg/h. Hence, we argue that treating the minimal average UO as a continuous predictor is a preferable strategy to define meaningful oliguria thresholds.

### Strengths and limitations

This study is the largest (> 15,000 patients) attempting to assess the relationship between minimal average UO and mortality. The sample includes the vast majority of patients admitted to a large multidisciplinary ICU. Hence, we were able to assess the influence of the type of admission (medical vs surgical) on the described relationship. In addition, the large sample size enabled to separate our cohort into a derivation and validation sub-cohorts and test the discrimination and calibration of our models. We used all UO data points across the entire patient stay. Hence, our data is representative of the entire patients’ admission. The inclusion of illness severity and comorbidity scores as well as age in our models enabled to control for the effect of important confounders on the relationship between UO and 90-day mortality. Finally, in contrast to previous studies of comparable size [12, 20], we have flexibly modeled UO as a continuous predictor of 90-day mortality.

Our study, however, has limitations. As a single center study, the external validity of our findings may be challenged. However, although relying on different types of statistical modeling, our findings are in line with previous smaller or un-adjusted studies and can be viewed as confirmatory. We have elected to restrict our analyses to convenience time windows of 3, 6, 12 and 24 h. Perhaps different time windows would have provided different results. However, due to the similarity of data obtained across all reported time windows, this appears unlikely. In addition, proposed time windows are similar to those currently utilized (except for the 3 h window) and appear clinically relevant and applicable. We have not considered other outcomes of potential importance such as increased sCr or need for RRT. Indeed, previous analyses have demonstrated that severe (stage 2 or 3) oliguria was an independent risk factor for mortality irrespective of sCr [2]. We believe that the demonstration of an impact on 90-day mortality is the most meaningful way to define oliguria. In addition, KDIGO criteria and the sCr criteria in particular were validated through their relevance on mortality. As this is a real-life study, we have not used an automated urimeter which might have improved the accuracy of the data collection. However, the low rate of missing values for UO suggests a high level of validity in our data. Lastly, UO was normalized to patients' body weight, a parameter known to be highly inaccurate in critical illness. This was minimized by the primary consideration of pre-admission weight which was available in 79.5% of the patients and imputed for the remaining 20.5% of patients. In addition, sensitivity analyses suggested that extreme weights did not impact our results [21].

### Implications for clinicians and policy makers

Our data provide an explanation for the perceived lack of relevance of oliguria as defined by a minimum average UO of less than 0.5 ml/kg/h for 6 h. Indeed, this definition appears to be too liberal and to include many patients for which oliguria has no prognostic implication. By considering UO as a continuous predictor of 90-day mortality, we propose to revise the current definition of oliguria and use 0.2 ml/kg/h for 6 h as a cut-off. Our data further suggests that such cut-off might be applied indifferently to patients with medical and surgical (emergent and elective) admissions despite the fact that these populations have very different baseline adjusted mortality rates.

Furthermore, we show that an increased mortality can already be identified when averaging UO over 3 h while using the same cut-off for oliguria (< 0.2 ml/kg/h). Using a time window of three hours to monitor urine output may thus allow early identification of patients at risk of excess mortality.

## Conclusions

In this large observational study, we confirmed that the current consensus threshold of 0.5 ml/kg/h over 6 h to define oliguria may be too conservative. A cut-off of 0.2 ml/kg/h over 3 or 6 h is supported by the data and should be considered in further definitions of oliguria.

## Supplementary Information

**Additional file 1.** Supplementary Table 1 and 2 and Figures S1–S12.

## Data Availability

The datasets used and/or analyzed as well as R code used in the current study are available from the corresponding author upon reasonable request.

## Abbreviations

AKI: Acute kidney injury
AU-ROC: Area under the receiver operator characteristic curve
ESRD: End-stage renal disease
ICU: Intensive care unit
IQR: Interquartile range
SAPS: Simplified acute physiology score
sCr: Serum creatinine
SD: Standard deviation
STROBE: Strengthening the reporting of observational studies in epidemiology
RRT: Renal replacement therapy
UO: Urinary output UO
