# Supplementary material for: Identification of an optimal threshold to define oliguria in critically ill patients: an observational study
Source: Crit Care. 2023 May 30;27:207. doi: 10.1186/s13054-023-04505-7 (PMC10228087; doi:10.1186/s13054-023-04505-7)
Supplement: Supplementary file 1 — Additional file 1. Supplementary Table 1 and 2 and Figures S1–S12. [file 13054_2023_4505_MOESM1_ESM.docx]

Supplementary Material to

**Identification of an Optimal Threshold to Define Oliguria in Critically-ill Patients: An Observational Study**

|  | **All patients**  **(n = 15'550)** | **Training set**  **(n = 12'440)** | **Validation set**  **(n = 3'110)** | **P-value** |
| --- | --- | --- | --- | --- |
| RRT during ICU stay, n (%) | 1154 (7.4) | 924 (7.4) | 230 (7.4) | 0.98 |
| Mechanical ventilation during ICU stay, n (%) | 9’595 (61.7) | 7’692 (61.8) | 1’903 (61.2) | 0.52 |
| Total duration of MV, median (IQR), hours^b^ | 35.8 (10.6, 122.6) | 35.3 (10.7, 123.9) | 37.3 (10.1, 118.6) | 0.71 |
| Noradrenaline during ICU stay, n(%) | 11’230 (72.2) | 8’973 (72.1) | 2’257 (72.6) | 0.64 |
| Follow-up length, median (IQR), months | 68.0 (34.0, 100.0) | 67.0 (34.0, 100.0) | 69.0 (34.0, 99.0) | 0.93 |
| ICU length of stay, median (IQR), days | 2.2 (1.1, 5.6) | 2.2 (1.1, 5.7) | 2.3 (1.1, 5.2) | 0.57 |
| Hospital length of stay, median (IQR), days | 12.8 (6.8, 23.4) | 12.8 (6.8, 23.6) | 12.8 (6.9, 22.9) | 0.75 |
| ICU mortality, n (%) | 1’715 (11.0) | 1’368 (11.0) | 347 (11.2) | 0.82 |
| Hospital mortality, n (%) | 2’293 (14.7) | 1’843 (14.8) | 450 (14.5) | 0.65 |
| 90 days mortality, n (%) | 2’859 (18.4) | 2’285 (18.4) | 574 (18.5) | 0.93 |
| 1 year mortality, n (%) | 3’453 (23.9) | 2’775 (24.0) | 678 (23.6) | 0.66 |
| 3 years mortality, n (%) | 3’630 (31.6) | 2’921 (31.7) | 709 (31.0) | 0.50 |
| 5 years mortality, n (%) | 3’192 (36.9) | 2’556 (37.0) | 636 (36.3) | 0.60 |
|  |  |  |  |  |

**Table S1: Patients’ outcomes**

Abbreviations: ICU: intensive care unit; RRT: renal replacement therapy; MV: mechanical ventilation; IQR: interquartile range. Missing values are presented in supplementary Table S2.

|  | **All Patients**  **n= 15’550** | **Derivation Cohort**  **n = 12’440** | **Validation Cohort**  **n =3’110** |
| --- | --- | --- | --- |
|  |  |  |  |
| **Demographics** |  |  |  |
| Gender | 0 (0) | 0 (0) | 0 (0) |
| Age at ICU admission | 0 (0) | 0 (0) | 0 (0) |
| Pre admission Body Weight | 3’188 (20.5) | 2’543 (20.44) | 645 (20.74) |
| Baseline creatinine | 324 (2.1) | 255 (2.1) | 69 (2.2) |
| **Comorbidities** |  |  |  |
| Charlson score | 49 (0.3) | 39 (0.3) | 10 (0.3) |
| Chronic kidney disease | 49 (0.3) | 39 (0.3) | 10 (0.3) |
| Hypertension | 49 (0.3) | 39 (0.3) | 10 (0.3) |
| Diabetes | 49 (0.3) | 39 (0.3) | 10 (0.3) |
| Heart failure | 49 (0.3) | 39 (0.3) | 10 (0.3) |
| Chronic obstructive pulmonary disease | 49 (0.3) | 39 (0.3) | 10 (0.3) |
| Myocardial infarction | 49 (0.3) | 39 (0.3) | 10 (0.3) |
| Chronic liver disease | 49 (0.3) | 39 (0.3) | 10 (0.3) |
| Cancer | 49 (0.3) | 39 (0.3) | 10 (0.3) |
| **ICU Admission characteristics** |  |  |  |
| Type of admission | 297 (1.9) | 244 (2) | 53 (1.7) |
| Main diagnosis | 352 (2.3) | 290 (2.3) | 62 (2) |
| Serum creatinine at ICU admission | 0 (0) | 0 (0) | 0 (0) |
| Mechanical ventilation first 24h | 0 (0) | 0 (0) | 0 (0) |
| Noradrenaline first 24h | 0 (0) | 0 (0) | 0 (0) |
| Modified SAPS II score | 0 (0) | 0 (0) | 0 (0) |
| **Outcomes** |  |  |  |
| RRT during ICU stay | 0 (0) | 0 (0) | 0 (0) |
| Mechanical ventilation during ICU stay | 0 (0) | 0 (0) | 0 (0) |
| Noradrenaline during ICU stay | 0 (0) | 0 (0) | 0 (0) |
| ICU length of stay | 0 (0) | 0 (0) | 0 (0) |
| Hospital length of stay | 0 (0) | 0 (0) | 0 (0) |
| ICU mortality | 0 (0) | 0 (0) | 0 (0) |
| Hospital mortality | 0 (0) | 0 (0) | 0 (0) |
| Mortality 3m | 0 (0) | 0 (0) | 0 (0) |
| Mortality 1yr | 1’116 (7.2) | 880 (7.1) | 236 (7.6) |
| Mortality 3yrs | 4’056 (26.1) | 3’235 (26) | 821 (26.4) |
| Mortality 5yrs | 6’889 (44.3) | 5’532 (44.5) | 1’357 (43.6) |

**Table S2: Missing values**

All values correspond to the number of patients with missing values among the specified datasets (%).


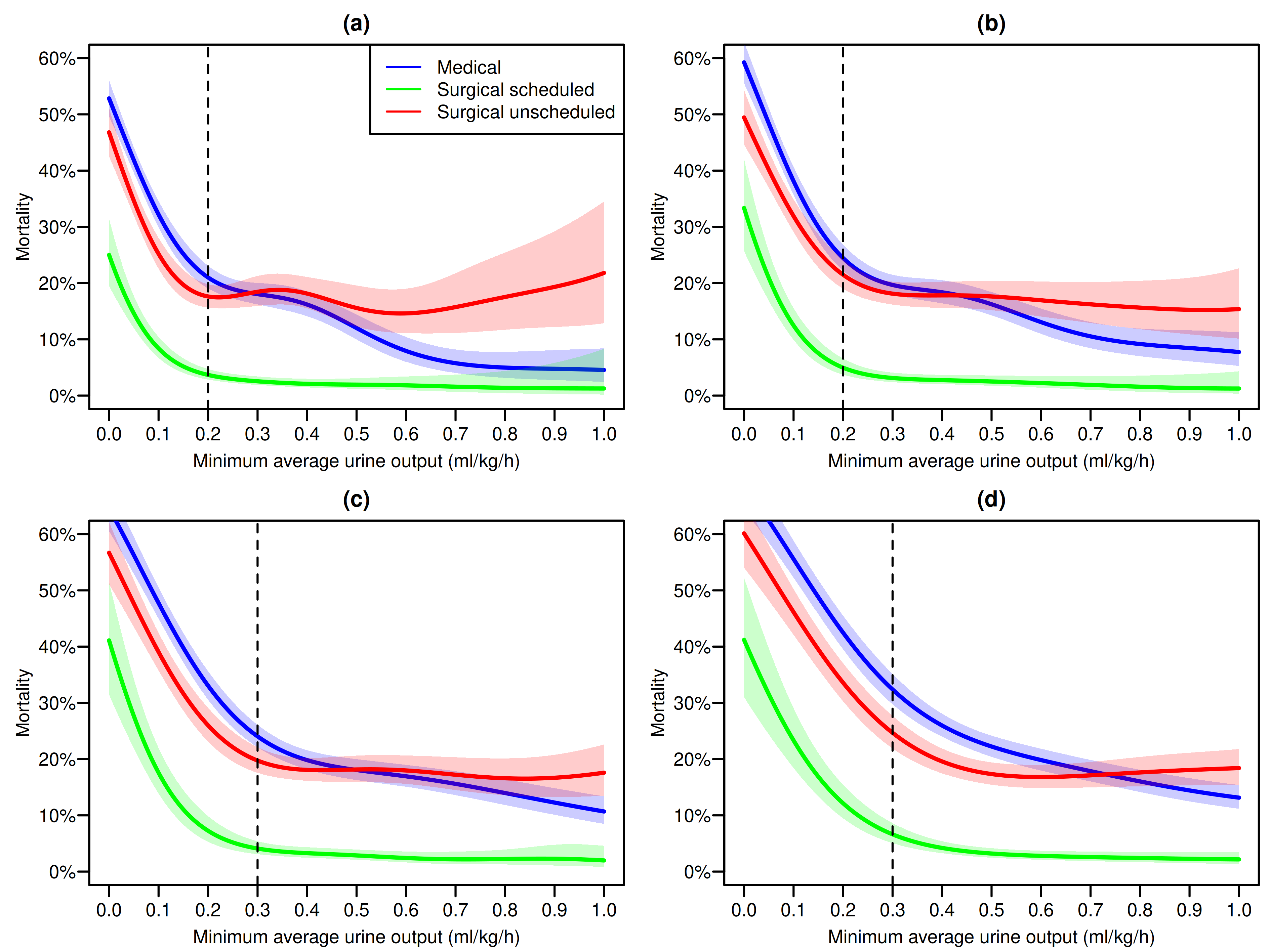


**Figure S1: Crude 90-day mortality as a function of the minimum average urine output for medical and scheduled/unscheduled surgical admissions for time windows of 3h (a), 6h (b), 12h (c) and 24h (d). Alternative to Figure 1 without confounding factors.**

Colored areas refer to 95% confidence intervals around the regression lines. Vertical dashed lines refer to thresholds below which the mortality increases rapidly.


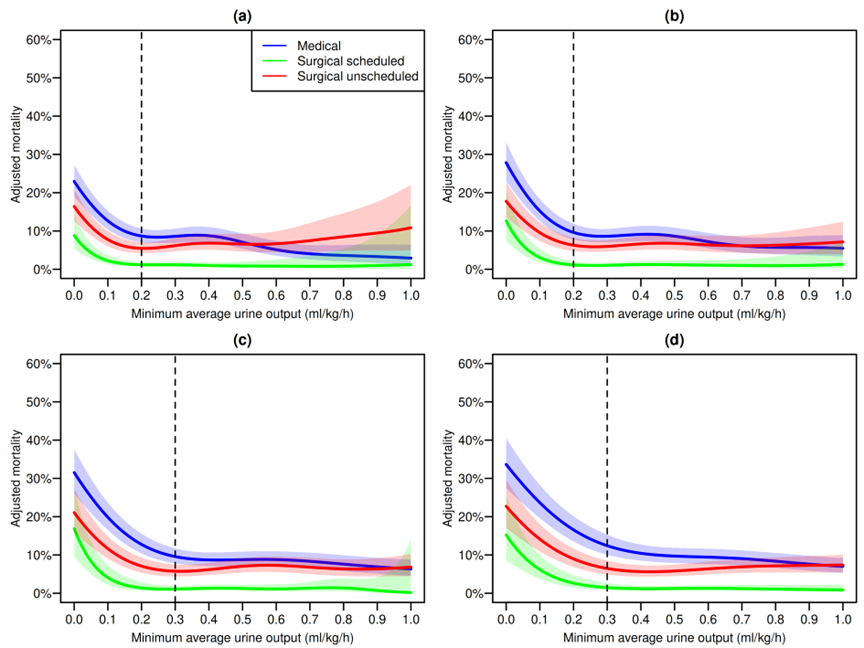


**Figure S2: Adjusted* 30-day mortality as a function of the minimum average urine output for medical and scheduled/unscheduled surgical admissions for time windows of 3h (a), 6h (b), 12h (c) and 24h (d). Alternative to Figure 1 with 30-day instead of 90-day mortality**

Colored areas refer to 95% confidence intervals around the regression lines. Vertical dashed lines refer to thresholds below which the adjusted mortality increases rapidly.

* Predictions are carried out for a fictive patient with continuous predictors fixed at their median value (i.e. 65 years old at ICU admission, corrected SAPS II score of 37 and Charlson index of 4).


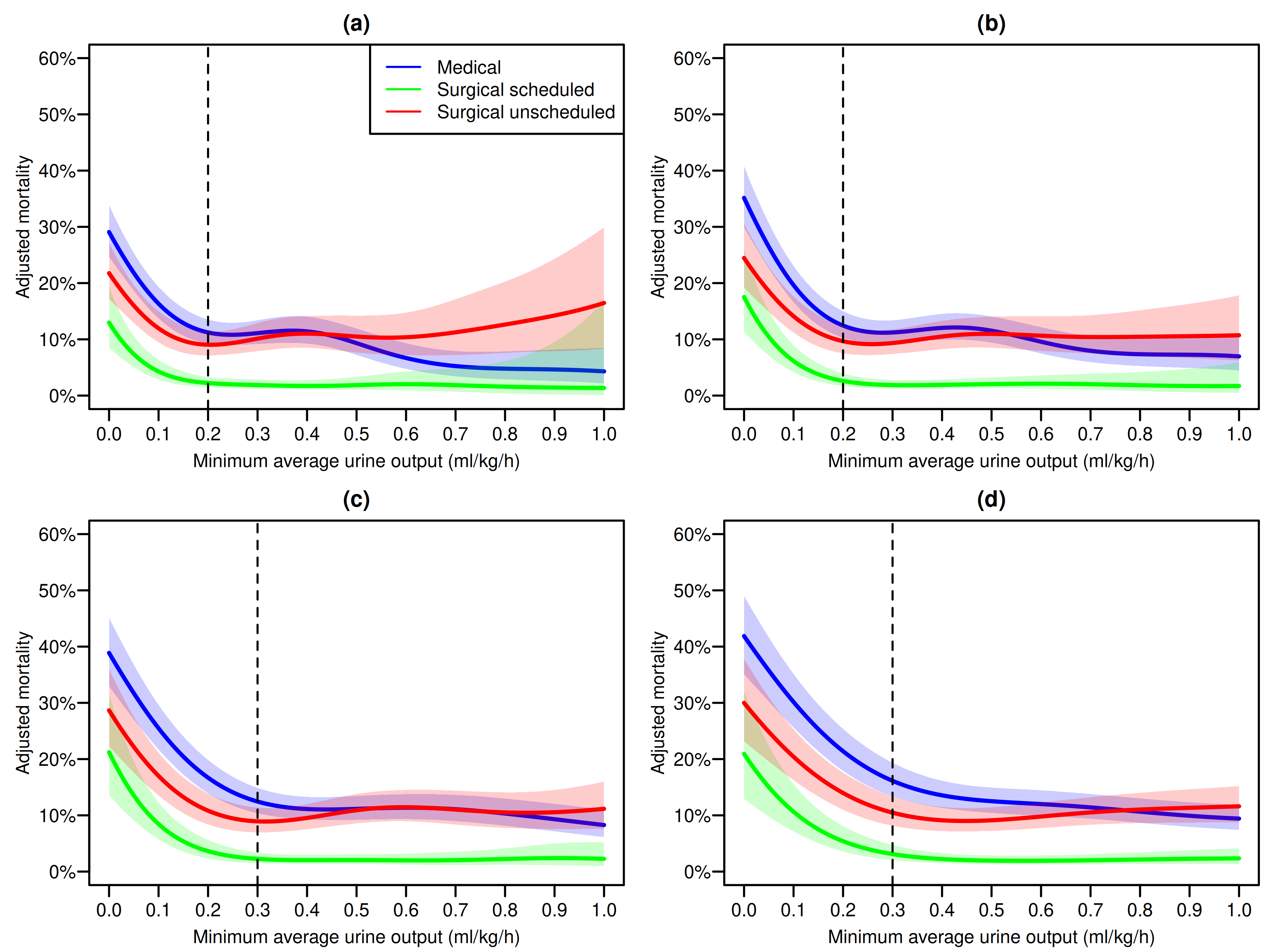


**Figure S3: Adjusted* 90-day mortality as a function of the minimum average urine output for time windows of 3h (a), 6h (b), 12h (c) and 24h (d). Alternative to Figure 1 considering only patients with a body weight >43kg and < 130kg.**

Data is stratified by type of admission (medical and scheduled/unscheduled surgical admissions). Colored areas refer to 95% confidence intervals around the regression lines. Vertical dashed lines refer to thresholds below which the adjusted mortality increases substantially.

* Predictions are carried out for a fictive patient with continuous predictors fixed at their median value (i.e. 65 years old at ICU admission, corrected SAPS II score of 37 and Charlson index of 4).


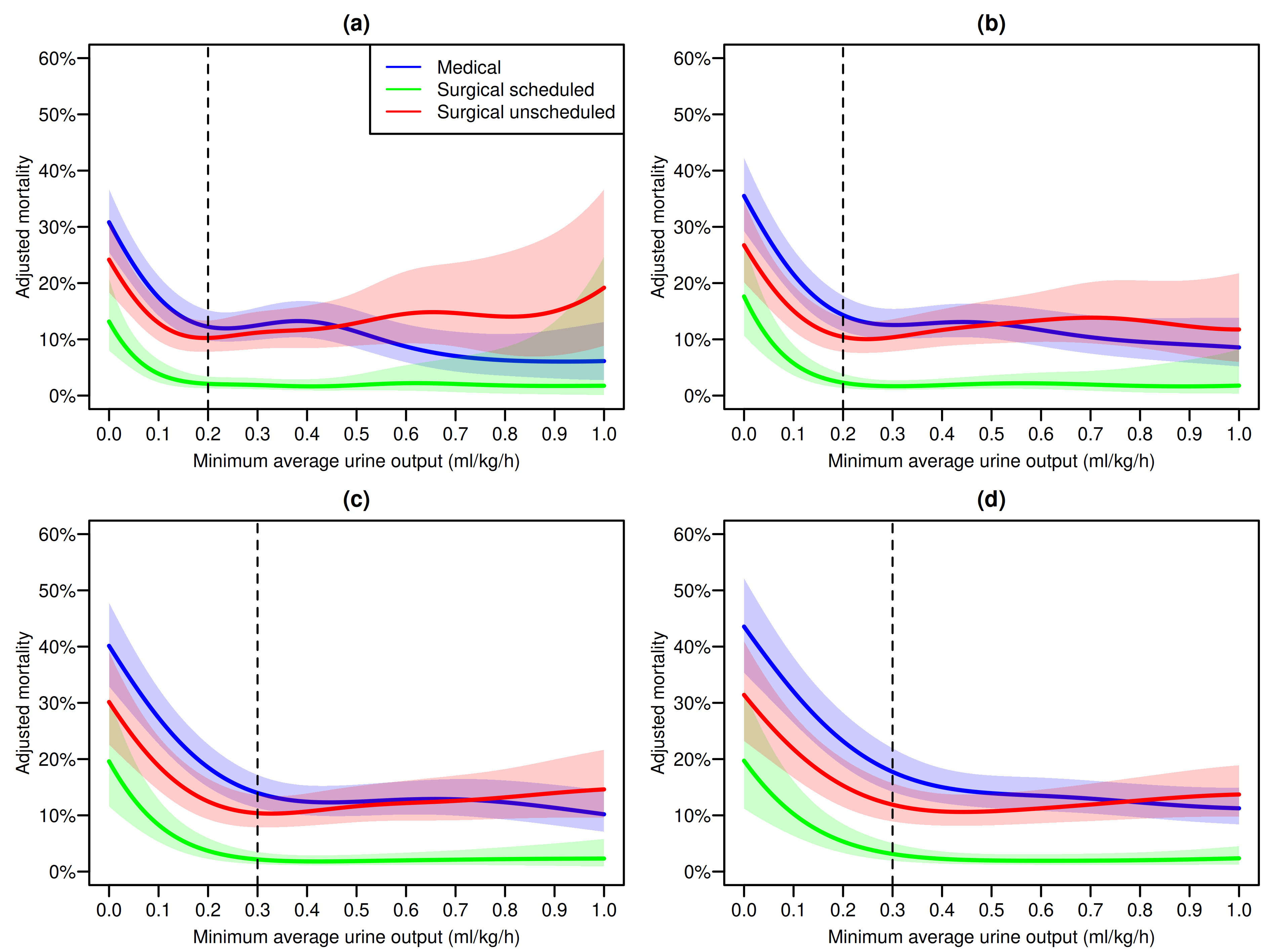


**Figure S4: Adjusted* 90-day mortality in males as a function of the minimum average urine output for time windows of 3h (a), 6h (b), 12h (c) and 24h (d). Alternative to Figure 1 considering only male patients.**

Data is stratified by type of admission (medical and scheduled/unscheduled surgical admissions). Colored areas refer to 95% confidence intervals around the regression lines. Vertical dashed lines refer to thresholds below which the adjusted mortality increases substantially.

* Predictions are carried out for a fictive patient with continuous predictors fixed at their median value (i.e. 65 years old at ICU admission, corrected SAPS II score of 37 and Charlson index of 4).


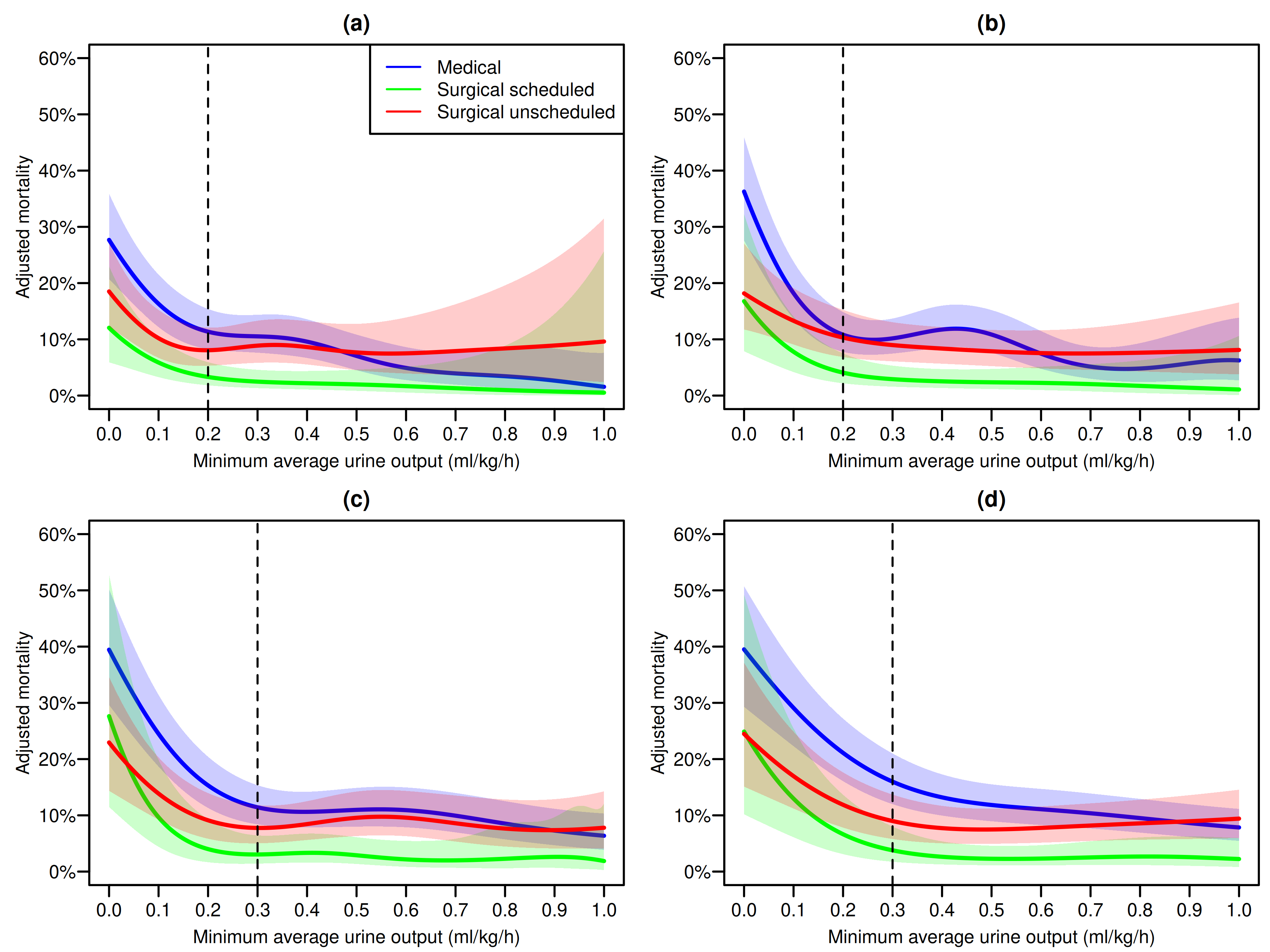


**Figure S5: Adjusted* 90-day mortality in females as a function of the minimum average urine output for time windows of 3h (a), 6h (b), 12h (c) and 24h (d). Alternative to Figure 1 considering only female patients.**

Data is stratified by type of admission (medical and scheduled/unscheduled surgical admissions). Colored areas refer to 95% confidence intervals around the regression lines. Vertical dashed lines refer to thresholds below which the adjusted mortality increases substantially.

* Predictions are carried out for a fictive patient with continuous predictors fixed at their median value (i.e. 65 years old at ICU admission, corrected SAPS II score of 37 and Charlson index of 4).


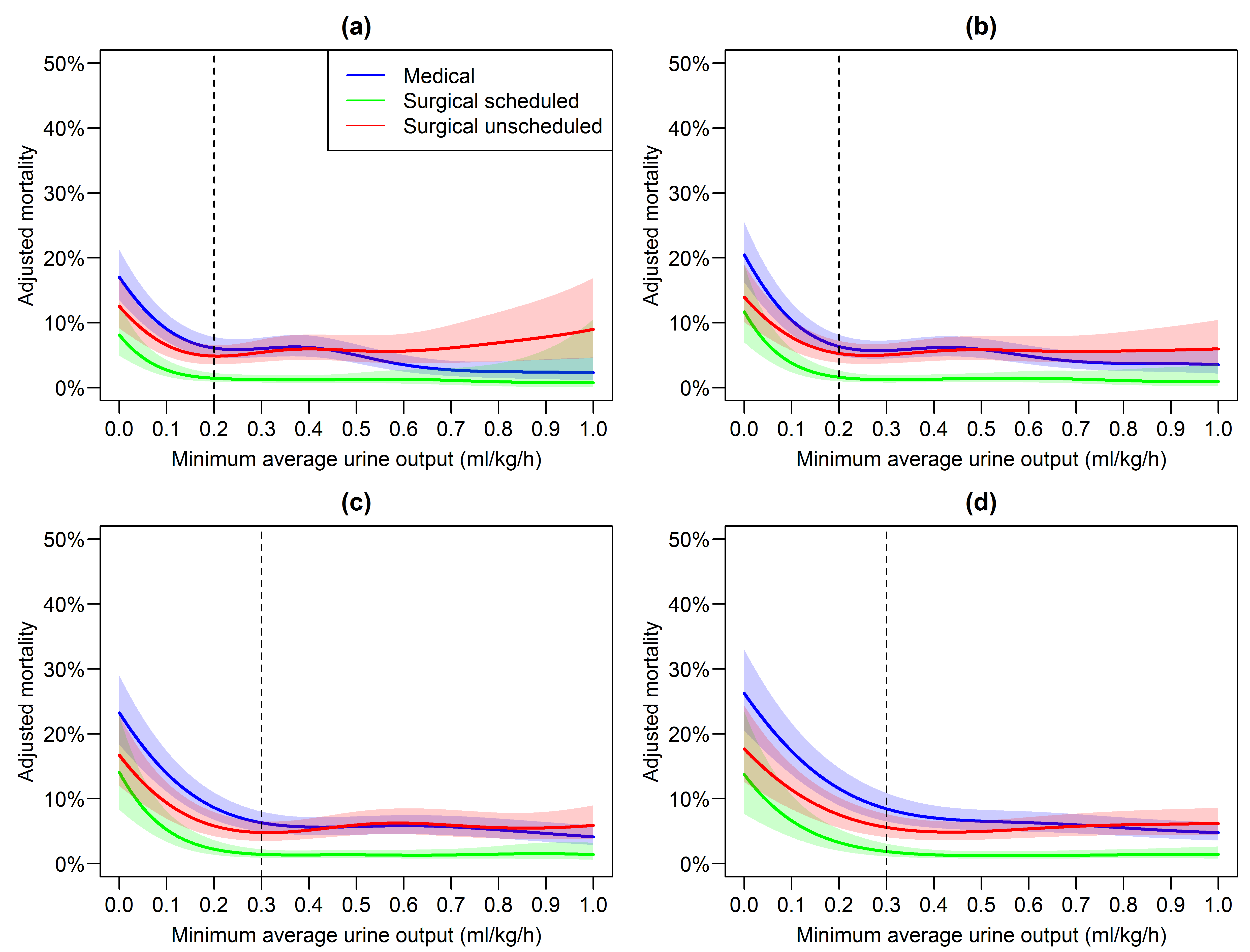


**Figure S6: Adjusted* mortality as a function of the minimum average urine output for medical and scheduled/unscheduled surgical admissions for time windows of 3h (a), 6h (b), 12h (c) and 24h (d).** **Alternative to Figure 1 with corrected SAPS II = 28 (Q1).**

Colored areas refer to 95% confidence intervals around the regression lines. Vertical dashed lines refer to thresholds below which the adjusted mortality increases rapidly.

* Predictions are carried out for a fictive patient with a corrected SAPS II score of 28 (first quartile) and other continuous predictors fixed at their median value (i.e. 65 years old at ICU admission and Charlson index of 4).


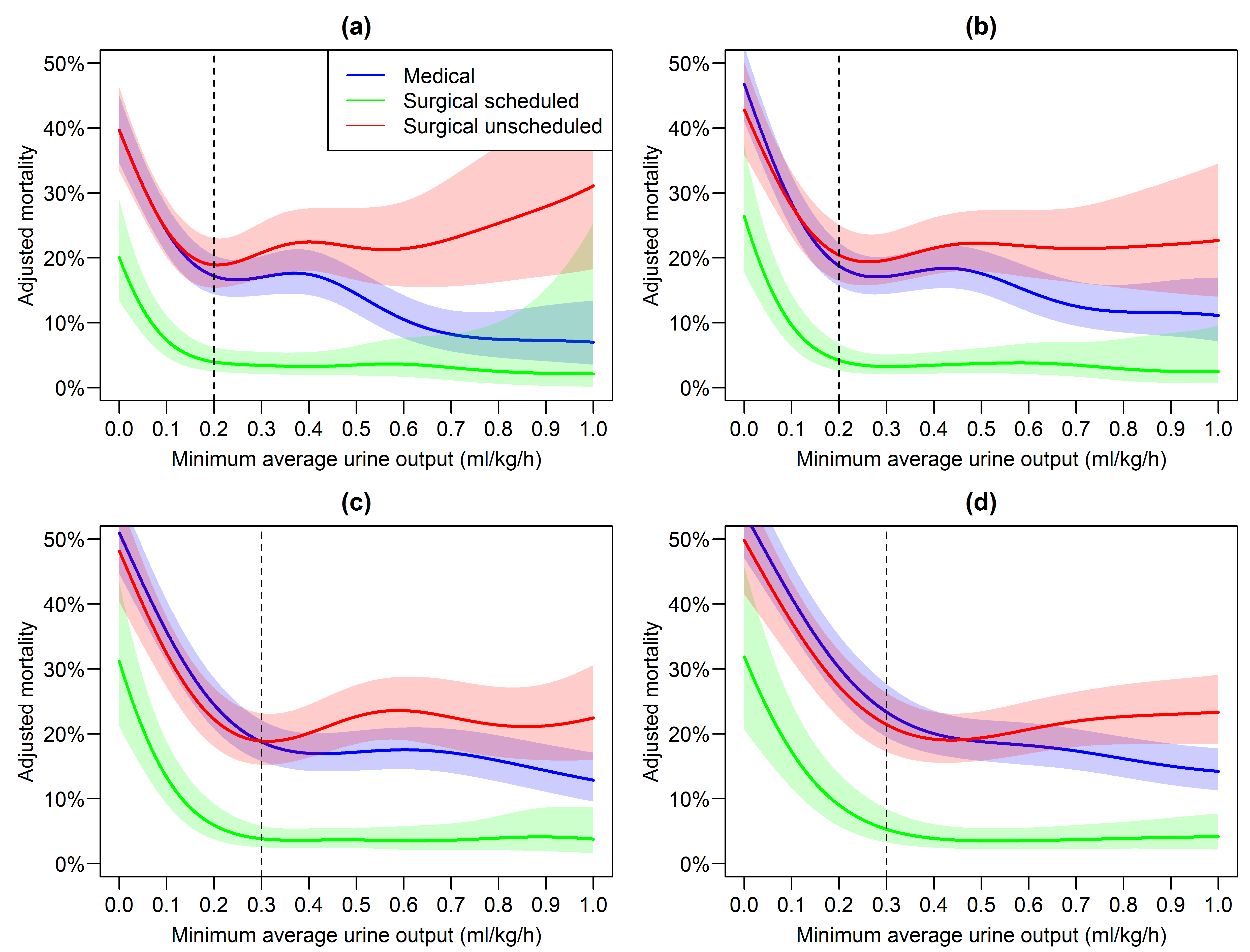


**Figure S7: Adjusted* mortality as a function of the minimum average urine output for medical and scheduled/unscheduled surgical admissions for time windows of 3h (a), 6h (b), 12h (c) and 24h (d). Alternative to Figure 1 with corrected SAPS II = 48 (Q3)**

Colored areas refer to 95% confidence intervals around the regression lines. Vertical dashed lines refer to thresholds below which the adjusted mortality increases rapidly.

* Predictions are carried out for a fictive patient with a corrected SAPS II score of 48 (third quartile) and other continuous predictors fixed at their median value (i.e. 65 years old at ICU admission and Charlson index of 4).


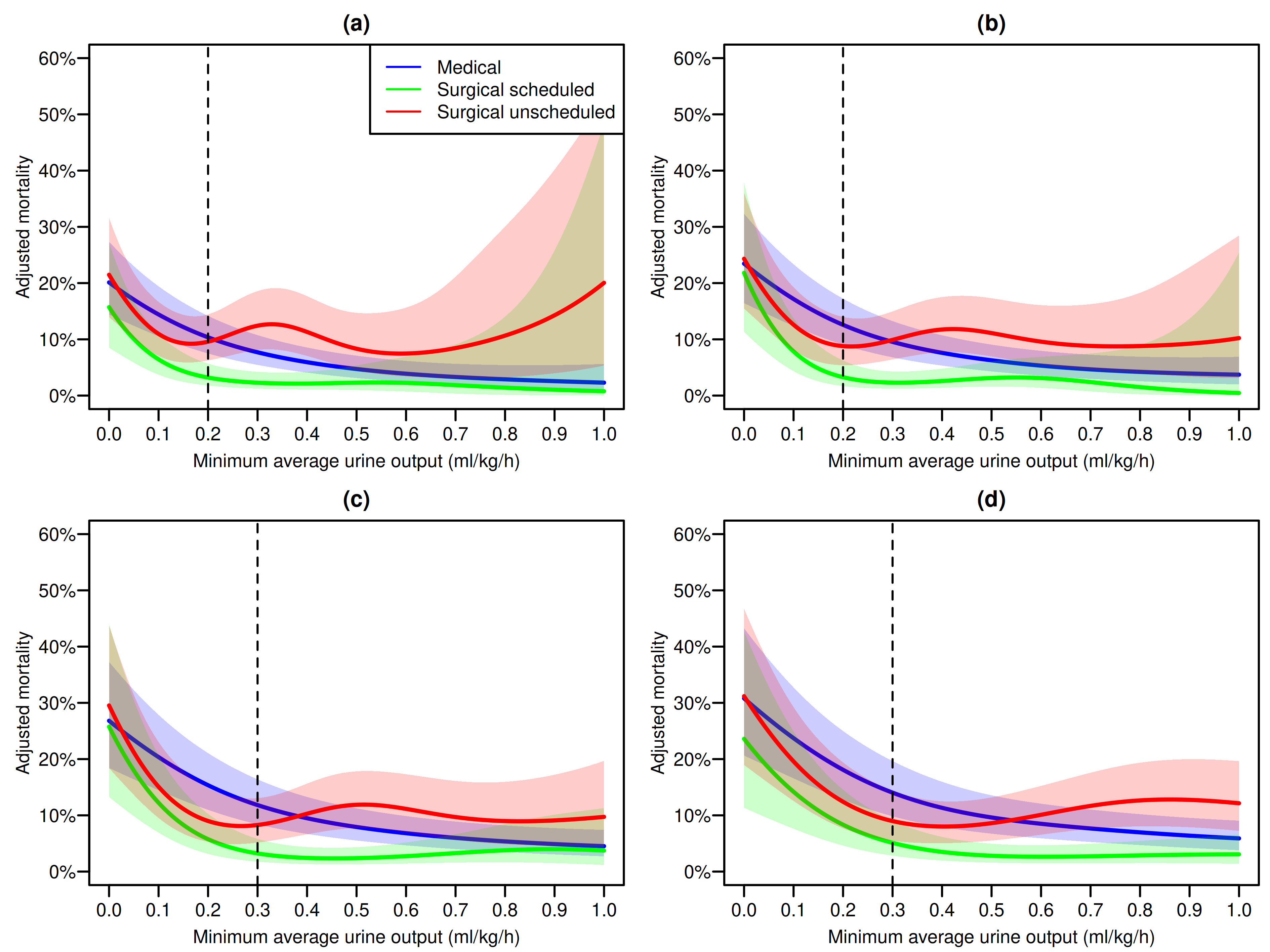


**Figure S8: Adjusted* 90-day mortality in patients admitted from 2010 to 2012, as a function of the minimum average urine output for time windows of 3h (a), 6h (b), 12h (c) and 24h (d). Alternative to Figure 1 considering only patients admitted between 2010 and 2012.**

Data is stratified by type of admission (medical and scheduled/unscheduled surgical admissions). Colored areas refer to 95% confidence intervals around the regression lines. Vertical dashed lines refer to thresholds below which the adjusted mortality increases substantially.

* Predictions are carried out for a fictive patient with continuous predictors fixed at their median value (i.e. 65 years old at ICU admission, corrected SAPS II score of 37 and Charlson index of 4).


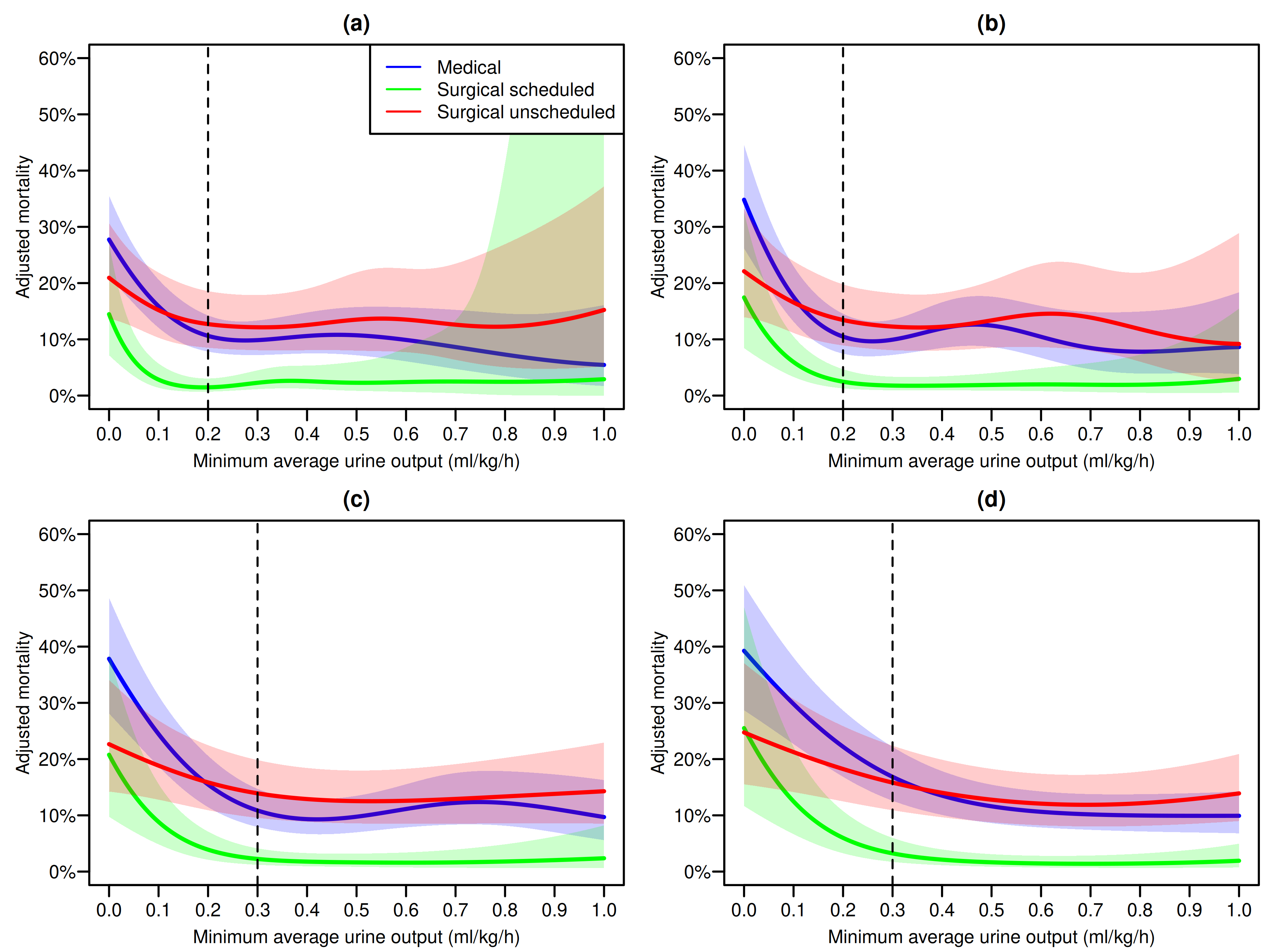


**Figure S9: Adjusted* 90-day mortality in patients admitted from 2013 to 2015, as a function of the minimum average urine output for time windows of 3h (a), 6h (b), 12h (c) and 24h (d). Alternative to Figure 1 considering only patients admitted between 2013 and 2015.**

Data is stratified by type of admission (medical and scheduled/unscheduled surgical admissions). Colored areas refer to 95% confidence intervals around the regression lines. Vertical dashed lines refer to thresholds below which the adjusted mortality increases substantially.

* Predictions are carried out for a fictive patient with continuous predictors fixed at their median value (i.e. 65 years old at ICU admission, corrected SAPS II score of 37 and Charlson index of 4).


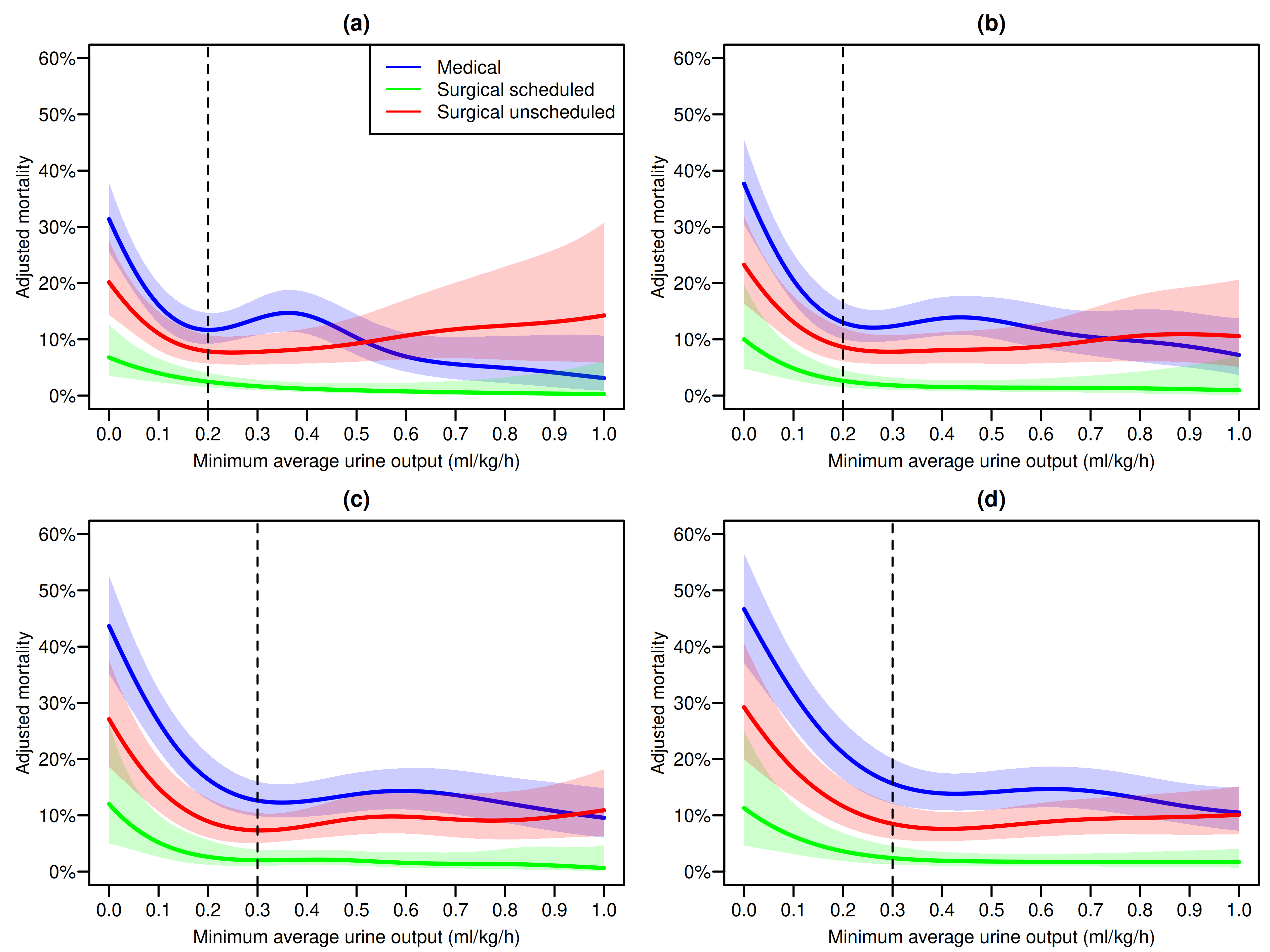


**Figure S10: Adjusted* 90-day mortality in patients admitted from 201**6 **to 2020, as a function of the minimum average urine output for time windows of 3h (a), 6h (b), 12h (c) and 24h (d). Alternative to Figure 1 considering only patients admitted between 2016 and 2020.**

Data is stratified by type of admission (medical and scheduled/unscheduled surgical admissions). Colored areas refer to 95% confidence intervals around the regression lines. Vertical dashed lines refer to thresholds below which the adjusted mortality increases substantially.

* Predictions are carried out for a fictive patient with continuous predictors fixed at their median value (i.e. 65 years old at ICU admission, corrected SAPS II score of 37 and Charlson index of 4).


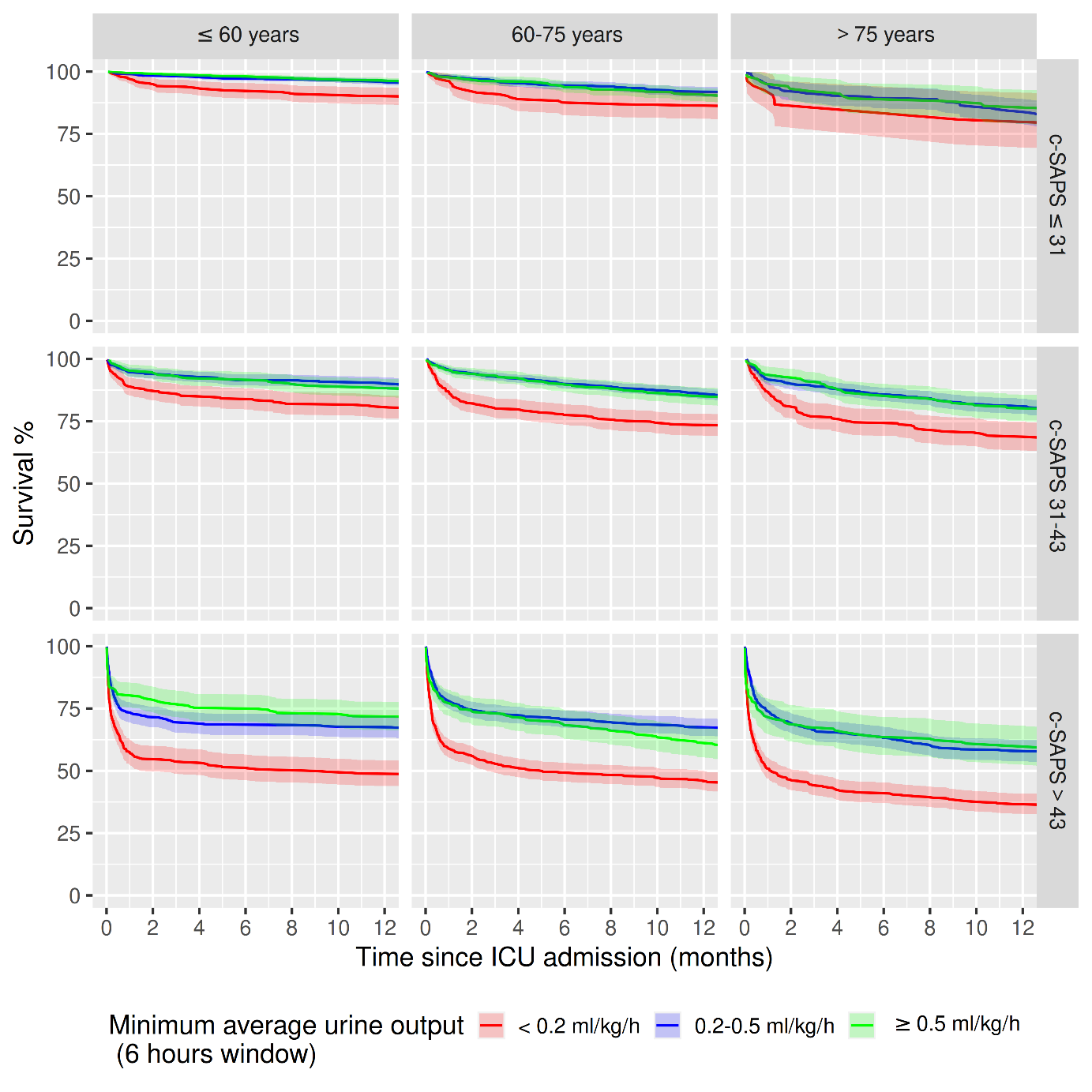


**Figure S11: Kaplan-Meier 12-month survival curves according to minimum average urinary output (6 hours windows).**

Data is stratified by tertiles of age and corrected SAPS score. Analyses are restricted to patients with available body weight (no imputation) n = 12’658

* Urine output corresponds to the minimum average urinary output over a period of 6 hours.


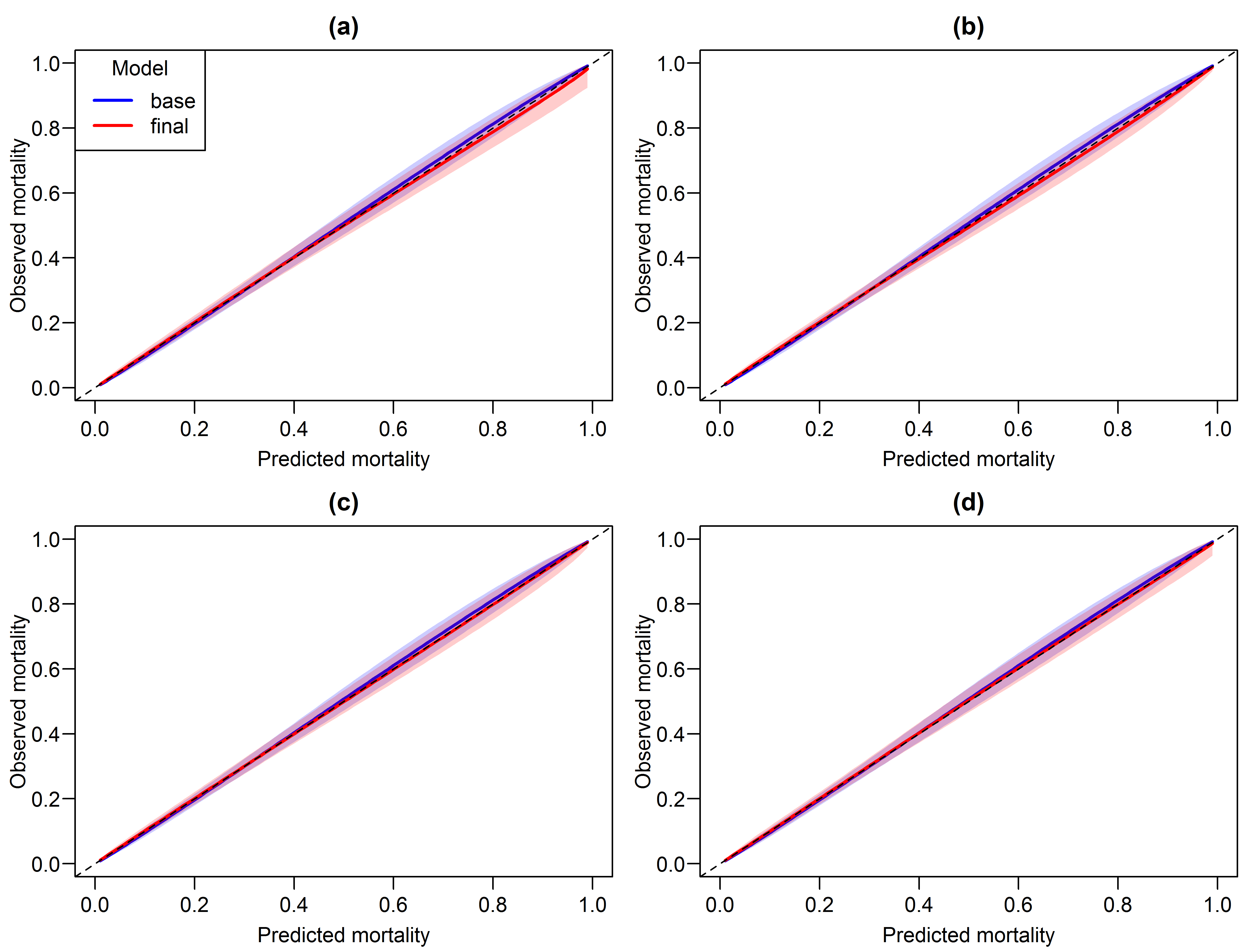


**Figure S12: Calibration belts for all patients in the validation set, for time windows of 3h (a), 6h (b), 12h (c) and 24h (d) for the base model (blue) and the final model.**

Colored areas refer to 95% pointwise confidence intervals for observed mortality.

### Methods supplement : Missing urinary output values management

In most cases, urine flow data were collected on an hourly basis. However, depending on the clinical workload, caregivers may not be able to enter the volume of urine excreted each hour into the electronic medical record, but instead enter the sum after a few hours, resulting in several missing hourly UO values preceding the corresponding entry. To consolidate the data, we divided this value by the number of adjacent missing values and spread the results across all these missing entries (e.g., the following sequence: ["NA","NA", "NA", 100] was replaced with [25, 25, 25, 25]). We did not account nor manage missing entries right before ICU discharge as they usually correspond to true missing values (vesical probe are usually removed when the patient has fully recovered). Remaining aberrant values (hourly UO above 1000ml) were replaced by the following hourly UO value.
